# *Anacardium occidentale* Bark as an Antidiabetic Agent

**DOI:** 10.3390/plants11192637

**Published:** 2022-10-07

**Authors:** Sofia Encarnação, Cristina De Mello-Sampayo, Belmira Carrapiço, Berta São Braz, Ana Patrícia Jordão, Conceição Peleteiro, Luís Catarino, Isabel B. Moreira da Silva, Luís F. Gouveia, Beatriz Silva Lima, Olga Silva

**Affiliations:** 1 Research Institute for Medicines (iMed.ULisboa), Faculty of Pharmacy, Universidade de Lisboa, 1649-003 Lisbon, Portugal; sofia.encarnacao@campus.ul.pt (S.E.); csampayo@ff.ulisboa.pt (C.D.M.-S.); icsilva@ff.ulisboa.pt (I.B.M.d.S.); lgouveia@campus.ul.pt (L.F.G.); beatrizlima@netcabo.pt (B.S.L.); 2 Centre for Interdisciplinary Research in Animal Health (CIISA), Faculty of Veterinary Medicine, Universidade de Lisboa, 1300-477 Lisbon, Portugal; belmira@fmv.ulisboa.pt (B.C.); bsaobraz@fmv.ulisboa.pt (B.S.B.); mcpelet@fmv.ulisboa.pt (C.P.); 3 Escola Universitária Vasco da Gama, 3020-210 Coimbra, Portugal; patricia.ramos.4080@gmail.com; 4 Centre for Ecology, Evolution and Environmental Changes (cE3c) & CHANGE—Global Change and Sustainability Institute, Faculty of Sciences, University of Lisbon, 1749-016 Lisbon, Portugal; lmcatarino@fc.ul.pt

**Keywords:** alpha-glucosidase inhibitors, *Anacardium occidentale*, antidiabetic, db/db mice, gallic acid, herbal medicines

## Abstract

*Anacardium occidentale* L. is used throughout the world to treat type 2 diabetes. In Portugal, a traditional herbal preparation made with stem bark of this species (AoBTHP) has been used for more than 30 years to treat this pathology. The AoBTHP was standardized on total phenolic content, and its hypoglycemic activity was assessed using db/db mice (*n* = 26) for 92 days. Three doses (40.2, 71.5, and 127.0 mg/kg/day, *per os*) were tested, and glibenclamide (5 mg/kg/day) was used as positive control. During the study, glycemia was measured under non-fasting or fasting states. In sequence, thin-layer chromatography bioautographic assays were used for the detection of possible alpha- and beta-glucosidase inhibitors. A significant hypoglycemic effect in fasting glycemia in days 31 and 57 was observed with the three tested doses. The 71.5 mg/kg and 127.0 mg/kg AoBTHPs significantly reduced non-fasting glycemia on day 24. The highest dose showed the most significant hypoglycemic effect. Gallic acid was identified as the major alpha- and beta-glucosidase inhibitor. The 127 mg/kg/day AoBTHP dose showed a greater glucose-lowering effect than glibenclamide. For the first time, a standardized AoBTHP was tested using an in vivo diabetes model, and its usage was preclinically validated for type 2 diabetes treatment. The hypoglycemic activity of an AoBTHP can be related to the presence of alpha- and beta-glucosidase inhibitors, such as gallic acid, but other mechanisms can also be involved.

## 1. Introduction

*Anacardium occidentale* L. is an *Anacardiaceae* Lindl. that is native to South America.It is cultivated in vast orchards in many tropical American, African, and Asian countries [1,2]. In traditional medicine, the use of *A. occidentale* has become widespread [3,4,5]. All around the world, dried *A. occidentale* stem bark (AoB) has been used to treat type 2 diabetes [6,7,8,9]. In Brazil, since the 19th century, it has been referred to as an official medicinal plant for diabetes [10]. In Portugal, an orally administered AoB traditional herbal preparation (THP), which is based on an AoB aqueous extract, has been used for more than 30 years as a daily treatment for type 2 diabetes [11]. 

According to the latest data of the WHO (2022), the global prevalence of diabetes among adults older than 18 years of age was 8.5% in 2014; this disease was the direct cause of 1.5 million deaths in 2019. In addition, the diabetes-related mortality rate increased by 13% in lower-middle-income countries [12].

Different pre-clinical in vivo studies have already been conducted to validate the glucose-lowering properties of AoB extracts (Table 1). All studies demonstrated the hypoglycemic activity of the AoB-tested samples [13,14,15,16,17,18]. Furthermore, due to the significant variability in the mode of preparation of the tested samples (solvent, type of extraction, ratio of medicinal plant to solvent), in the selected in vivo animal model (e.g., streptozotocin vs. fructose 25%), and in the duration of the studies, no comparison can be made to correlate chemical composition and effectiveness of the tested AoB preparations (Table 1).

Phytochemical studies conducted by different authors allowed for the identification of different chemical classes of constituents on AoB, including phenolic compounds (flavonoids, tannins, coumarins, and phenol acid derivatives) and terpenoids (saponins) [14,16,19,20,21,22]. Stigmast-4-en-3-one was isolated from a hypoglycemic hexane AoB extract [14]. More recently, gallic acid, catechin, epicatechin, and epigallocatechin were identified using high-performance liquid chromatography with an ultraviolet diode array detector in the ethyl acetate phase of an AoB acetone extract [23]. N-nonadecanoyl-*β*-sitosterol, gallic acid, and tanacetene were also isolated in an AoB methanolic extract [24]. However, no studies concerning the identification of the main compounds of THP based on AoB aqueous extracts were made by others.

Our team also conducted some phytochemical and pharmacological studies on this medicinal plant, using raw plant material from Guinea-Bissau, an African Portuguese-speaking country [21,25]. In that country, two main types of *A. occidentale* are recognized on the basis of the red or white false fruit color, and both are singly used to prepare AoB herbal medicines to treat type 2 diabetes. Phenol derivatives, mainly hydrolyzable and condensed tannins, were identified as major constituents of these raw materials, and gallic acid was identified as a major compound in these herbal medicines via thin layer chromatography (TLC) [25]. In sequence, the AoBTHPs were standardized on the basis of their total phenolic content expressed as milligrams of gallic acid equivalents (GAE) per gram of dried AoB [21], following the requirements of the World Health Organization [26]. These AoBTHPs were equally the target of an in vivo preclinical safety assessment, where they were studied for their general toxicity using a repeated dose toxicity test. No treatment-related signs of toxicity were observed in mice with doses up to 402 mg/kg of both AoBTHPs. Furthermore, the genotoxicity was evaluated in mice using a micronucleus test and a comet assay, and both AoBTHPs revealed a lack of genotoxic potential [21]. 

The main objectives of the present study were to determine the in vivo antidiabetic activity of a standardized Portuguese AoBTHP using the red type of CSB, and its mechanisms of action, namely, its *α*- and *β*-glucosidase inhibitory activities.

## 2. Results

### 2.1. HPLC-UV/DAD Analysis

**Figure 1:**
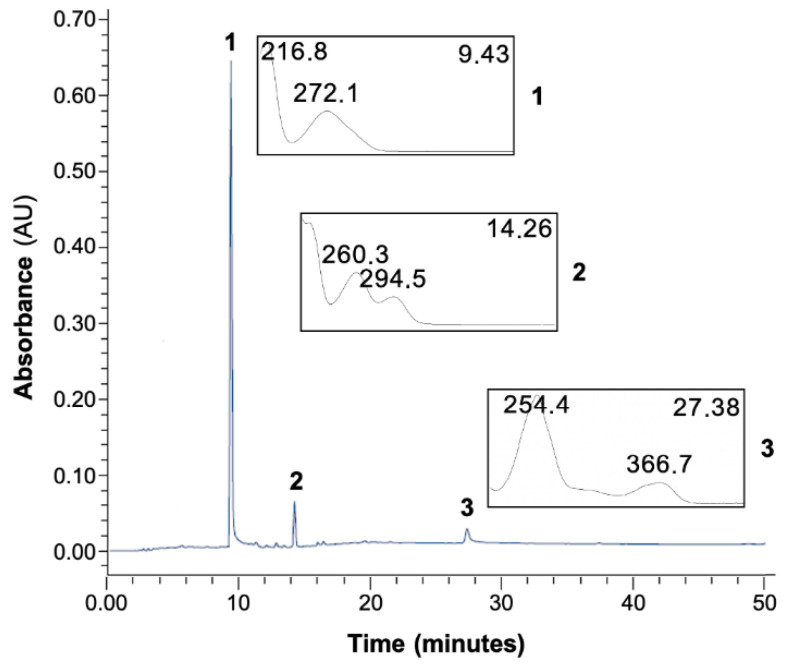
Representative HPLC-UV/DAD chromatographic profile for the AoBTHP. 1: gallic acid; 2: protocatechuic acid; 3: ellagic acid.

A comparison of the retention time and ultraviolet photodiode array (UV/DAD) absorption spectrum of each standard with the peaks obtained in the chromatogram for the AoBTHP shows that the peaks labeled 1, 2, and 3 in Figure 1 correspond to gallic acid, protocatechuic acid, and ellagic acid, respectively. Identification of these compounds was also confirmed using co-chromatography data.

### 2.2. Extract Standardization

The determined value of the standardization of the Portuguese AoBTHP based on its total phenolic content was 31.39 ± 0.50 mg GAE/g AoB.

### 2.3. In Vivo Evaluation of the Hypoglycemic Activity

#### 2.3.1. Clinical Signs

No adverse treatment-related abnormalities in the behavior of mice were detected during the study. In the group that was treated with the highest dose of the AoBTHP (127.0 mg/kg) and in the positive control group, one animal died on day 2 (animal 2 and animal 1, respectively), due to administration error that was confirmed via necropsy.

#### 2.3.2. Body Weight

The weekly mean body weight data are shown in Table 2. No statistically significant differences (*p* > 0.05) were found between groups in the beginning and at the end of the study concerning this parameter. With time, the mean body weight changed in all groups. 

The mean body weight decreased from week 0 to week 3, but after that, all groups increased their body weight over time. A significant increase was observed in the negative control group, between the final vs. initial body weight. Nevertheless, no statistically significant differences (*p* > 0.05) on that parameter were observed in the positive control and treatment groups.

#### 2.3.3. Food and Water Consumption

**Figure 2:**
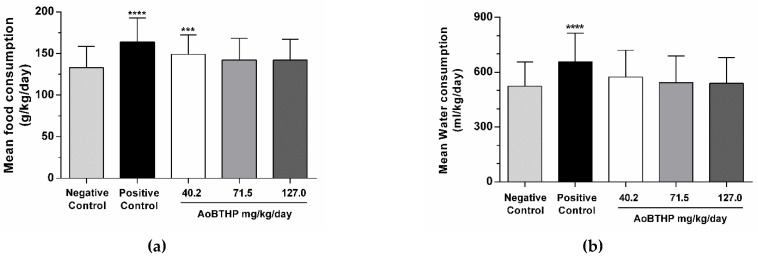
(**a**) Mean food consumption relative to average body weight per group of animals during the study (g/kg bw/day); (**b**) mean water consumption relative to average body weight per group of animals during the study (ml/kg bw/day). *** *p* < 0.001 vs. negative control; **** *p* < 0.0001 vs. negative control.

The mean food consumption (food consumption divided by average body weight) data are presented in Figure 2a. Statistically significant differences in food consumption were observed between the 40.2 mg/kg AoBTHP (*p* < 0.001) and the positive control (*p* < 0.0001), compared to the negative control group. No statistically significant differences (*p* > 0.05) were observed between the other groups and the negative control.

The mean water consumption (water consumption divided by average body weight) data are presented in Figure 2b. There were statistically significant differences (*p* < 0.0001) in water consumption between control groups. However, no statistically significant differences were observed concerning these parameters between the AoBTHP treatment groups and the negative control group.

#### 2.3.4. Biochemical Parameters

The non-fasting glycemia values of the blood samples collected during the study in days 0, 24, 43, and 71 are presented in Table 3. The 127.0 mg/kg AoBTHP treatment group was the only one that registered significant differences on non-fasting glycemia when compared to the vehicle-treated animals (negative control group) in day 43 (*p* < 0.05). In fact, there were no statistically significant differences (*p* > 0.05) between non-fasting glycemia values in the glibenclamide treated animals (positive control group) and in the groups treated with the lowest doses of the AoBTHP (40.2 mg/kg and 71.5 mg/kg), when compared to the negative control group. However, statistically significant differences (*p* < 0.05) on non-fasting glycemia were observed within the positive control and the 71.5 mg/kg AoBTHP treatment groups between day 0 and day 24. Within the 127.0 mg/kg AoBTHP treatment group, statistically significant differences were observed in non-fasting glycemia between day 0 and days 24 (*p* < 0.01) and 43 (*p* < 0.05).

The values of glycemia in the fasting state registered in days 8, 31, and 57 of the study are presented in Table 4. By study days 31 and 57, the glibenclamide treated animals, as well as those treated with the AoBTHP (40.2, 71.5 and 127.0 mg/kg), showed significantly lower fasting glycemia as compared to the vehicle-treated animals. Furthermore, only within the 127.0 mg/kg AoBTHP treatment group, statistically significant differences were observed on days 37 (*p* < 0.0001) and 57 (*p* < 0.01) when compared with the day 8 fasting glycemia data. 

**Figure 3:**
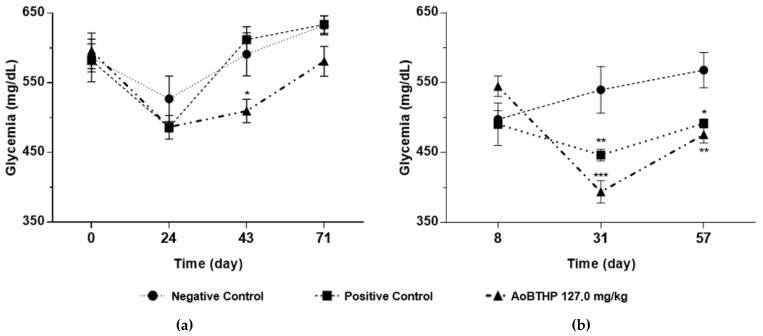
(**a**) Non-fasting glycemia values; (**b**) fasting glycemia values of control groups and 127.0 mg/kg AoBTHP. * *p* < 0.05 vs. negative control, ** *p* < 0.01 vs. negative control, *** *p* < 0.0001 vs. negative control.

The differences in mean glycemia values between fasting and non-fasting states of both control groups, and the AoBTHP highest dose treatment group, are depicted in Figure 3. The lowest non-fasting and fasting glycemia values were registered on days 24 and 31, respectively.

The biochemical parameters assessed at the end of the study (alanine transaminase, aspartate transaminase, cholesterol, glycemia, serum creatinine, serum urea, and triglycerides) are shown in Table 5. No statistically significant differences (*p* < 0.05) were observed concerning these parameters among groups.

#### 2.3.5. Organ Weights

The relative organ weights are presented in Table 6. No statistically significant differences (*p* > 0.05) in relative heart, kidney, liver, pancreas, and spleen weights were found among the studied animal groups.

#### 2.3.6. Histological Analyses

**Figure 4:**
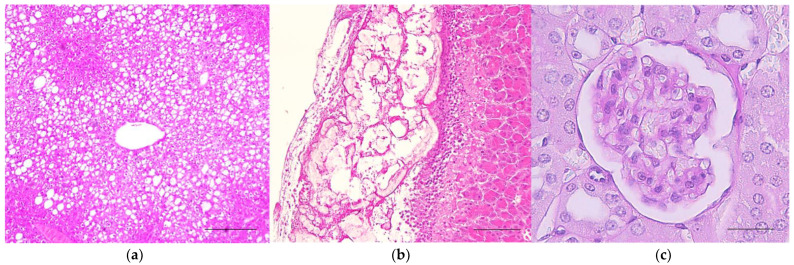
Histopathological analysis in tissues. (**a**) Liver section revealing more pronounced hepatocyte vacuolation in the centrilobular region from a mouse treated with 40.2 mg/kg AoBTHP (40× magnification, hematoxylin, and eosin staining). (**b**) Pancreas section showing foci of necrotic adipocytes, with discrete inflammatory infiltration contiguous with areas of neutrophilic cellular infiltration, at the periphery of a pancreatic lobe from a mouse treated with 127.0 mg/kg AoBTHP (100× magnification, hematoxylin and eosin staining). (**c**) Glomerular section with a basement membrane with normal thickness and mesangial cell nuclei in normal number, from a mouse treated with 127.0 mg/kg AoBTHP (400× magnification, periodic acid-Schiff staining). Scale bars: A = 200 µm; B = 100 µm; C = 20 µm.

The histological analyses showed some structural alterations in all groups of animals, suggesting non-relation to the treatments. The liver sections revealed vacuolar hepatopathy, mostly centrilobular (Figure 4a). Pancreatic tissue presented no relevant changes except for a few cases of acute pancreatitis and/or steatonecrosis (Figure 4b). The kidney tissue generally appeared unaltered (Figure 4c). However, cases of acute pyelonephritis and/or suppurative interstitial nephritis were observed. No glomerular deposits were seen with periodic acid-chiff staining. The heart, intra-abdominal fat, and spleen showed no changes. 

### 2.4. In Vitro Evaluation of the Hypoglycemic Activity

#### 2.4.1. α-Glucosidase Inhibitory Assay

In this assay, the substrate *p*-nitrophenyl-*α*-D-glucopyranoside was hydrolyzed by α-glucosidase, forming a yellow-colored product; its absorbance was measured with an analyzer microplate reader. The IC_50_ values were 1.15 ± 0.00 mg/mL of AoB and 43.97 ± 0.21 mg/mL to the acarbose.

#### 2.4.2. Detection of α- and β-Glucosidase Inhibitors by Bioautography

With bioautography, after TLC plates were sprayed with the enzyme solutions, pale yellowish *α*-glucosidase inhibitory zones with a retention factor (R*_f_*) ranging from 0.62 to 0.78, and *β*-glucosidase inhibitory zones with a R*_f_*ranging from 0.58 to 0.75, were observed. 

The chromatographic co-elution of gallic acid standard and the AoBTHP confirmed that this compound was the main *α*- and *β*-glucosidase inhibitor in the tested sample, showing a blue fluorescence, and an R*_f_* of 0.68.

## 3. Discussion

Diabetes mellitus is a group of chronic metabolic diseases resulting from a relative or complete lack of insulin secretion by *β*-cells of the pancreas, and/or the decreased response to insulin by target tissues [27]. It is characterized by hyperglycemia and disturbances of carbohydrate, fat, and protein metabolism; these symptoms lead to severe complications and organ failure over time [28]. Type 2 diabetes is the predominant form, accounting for more than 95% of total cases. Its onset classically occurs in middle-aged and elderly patients, with the incidence in early adulthood and childhood increasing [12,29]. Factors such as obesity, sedentary lifestyle, and genetic predisposition play a critical role in the development of this type of diabetes [30,31], which results from insulin resistance in peripheral tissues, and insulin secretory defects [32]. The management of patients with type 2 diabetes may include diet and lifestyle modifications, frequently in association with oral hypoglycemic drugs and, in some cases, subcutaneous insulin therapy [27,33,34].

Despite the increased availability in the last decade of innovative therapies for type 2 diabetes, there is still less well-understood patient variability in the response to those treatments to achieve appropriate glycemic control. Furthermore, patients not only vary in the therapeutic response, but also vary in the potential for developing adverse reactions to treatment. The aspects that are associated with increasing age motivate the search for additional therapeutic approaches for the disease [35]. Currently, species of plants used in traditional medicine for managing diabetes have become the target of numerous studies for the research and development of new antidiabetic drugs [36,37]. Galegine, phenolic compounds (such as anthocyanins and flavonoids), and pycnogenol are examples of plant-derived compounds with antidiabetic activity [38]. Medicinal plants can be an alternative therapeutic approach to treating type 2 diabetes, but it is essential to increase the number of studied species and adequately conducted research [39]. The verification of their effectiveness and mode of action is a mandatory step in the development of a formulation that can be explored and reproduced by the medicinal plant industry [40].

The db/db mouse model was chosen for this study because it is a well-studied and recognized model in research on hypoglycemic activity [41]. This model mimics overt type 2 diabetes in humans so that we can translate scientific evidence from this animal research to humans [42].

The db/db mouse is an animal that is homozygous for the spontaneous diabetes mutation (Leprdb) that becomes obesity at approximately three to four weeks of age. This animal model for severe type 2 diabetes is characterized by the development of metabolic syndrome, where hyperglycemia, hyperinsulinemia, and obesity coexist [43]. The severity of disease in the db/db mouse leads to an uncontrolled rise in glycemia, severe depletion of insulin-producing *β*-cells of the pancreatic islets, obesity at 3 to 4 weeks, and death by 10 months of age [44]. Elevations in plasma insulin begin at 10 to 14 days, and the animals primarily become hyperglycemic at 6 to 8 weeks and, posteriorly, markedly hyperglycemic at 4 to 6 months. Inevitably, exogenous insulin fails to control glycemia levels, and gluconeogenic enzyme activity increases [32].

The AoB hypoglycemic activity was previously assessed in streptozotocin-induced diabetic rats [15,16,17], healthy dogs [13,14], and fructose-diabetic rats [18] which do not mimic the development of type 2 diabetes as well as the db/db mouse model chosen by our team. Additionally, some unusual toxic solvents, hexane [13,14] and methanol [17,18], were used as extraction solvents, and the prepared extracts were not chemically standardized. Furthermore, all other studies conducted with AoB had shorter durations than our 92-day study, ranging from 2 h to 28 days (Table 1).

As stated before, to evaluate the hypoglycemic potential of the AoBTHP, an oral antidiabetic drug was used as positive control: glibenclamide. This drug belongs to the sulfonylureas class, and acts by binding to ATP-sensitive potassium channel receptors on the pancreatic cell surface, which results in a reduction in potassium conductance and depolarization of the membrane. This depolarization stimulates calcium ion influx through voltage-sensitive calcium channels, raising intracellular concentrations of calcium ions and, consequently, inducing insulin secretion [45]. Thus, glibenclamide is most effective in the early stages of type 2 diabetes when the number of functioning *β*-cells is still relatively unaffected [46]. However, glycemic control is inevitably lost over time with the glibenclamide treatment; therefore, the introduction of combination therapy is necessary to regain control and prolong the time before beginning exogenous insulin therapy [27].

In our study, both glibenclamide-treated animals (positive control group), and AoBTHP treatment groups appeared to have significantly lower fasting glycemia values when compared to untreated animals (negative control group) by study days 31 and 57. This activity was dose-dependent and more pronounced with the highest dose of the AoBTHP. As predicted by Brunton et al. [27], initially the glibenclamide was effective. However, the glycemic levels relevantly increased as the db/db mice aged, and severe type 2 diabetes appeared to develop. The same behavior was observed with the tested AoBTHP, suggesting an age-dependent hypoglycemic activity due to deteriorating glucose metabolism with increasing age [44]. Within the 127.0 mg/kg AoBTHP treatment group, significant differences on fasting glycemia still registered between day 8 and days 31 and 57, which is in line with a dose-related potency.

Regarding the non-fasting glycemia, we observed a hypoglycemic effect within the positive control, the 71.5 mg/kg AoBTHP, and the 127.0 mg/kg AoBTHP treatment groups between day 0 and days 24. Furthermore, within the 127.0 mg/kg AoBTHP treatment group, a reduction in non-fasting glycemia values was also observed between day 0 and day 43. 

Thus, the 127.0 mg/kg AoBTHP treatment group presented the highest hypoglycemic effect in both fasting and non-fasting glycemia, showing a more potent glucose-lowering effect than glibenclamide. 

At the beginning of the study, we ensured no significant differences in body weight between groups. As expected for the db/db mouse model, the negative control showed a statistically significant increase in weight during the study. However, no significant body weight changes were observed in the positive control group and the AoBTHP treatment groups between the beginning and the end of the study, which could be due to some treatment effect.

During the study, the mean water and food consumptions were statistically significantly m in the positive control group compared to the negative control group. No change in food consumption was observed in the highest doses of AoBTHP-treated animals.

It is important to note that no significant changes (*p* > 0.05) were observed in biochemical parameters (ALT, AST, cholesterol, serum creatinine, serum urea, and triglycerides) that were assessed after the necropsies. Furthermore, no relevant morphological alterations were observed in the morphology of the assessed organs (heart, intra-abdominal fat, kidneys, liver, pancreas, and spleen) that could be related to the AoBTHP treatment. These results agree with the data obtained by our team in a repeated dose toxicity test performed with the same formulation [21].

In our study, *α*- and *β*-glucosidase enzymes were investigated as potential targets for AoB using bioautographic assays [45]. The results suggest that the hypoglycemic potential of the AoBTHP may be at least in part related to its inhibitory effects on these enzymes, as it has already been reported with *A. occidentale* leaf [47] and nutshell liquids [48]. Anacardic acids have been pointed out as the main compounds responsible for the hypoglycemic activity of *A. occidentale* extracts [49]; however, according to the phytochemical screening previously performed, these compounds were almost nonexistent in the tested AoBTHP [25]. Nevertheless, gallic acid was identified by our team as the main compound that was responsible for the *α*- and *β*-glucosidase inhibitory activity of the AoBTHP. These *α*-glucosidase inhibitory properties of gallic acid had already been reported by other authors [50,51].

Furthermore, in a previous study performed by our team, a DPPH assay revealed that the AoBTHP exhibited concentration-dependent antioxidant activity, with IC_50_ values of 180.7 ± 6.7 μg/mL [21]. Thus, it was concluded that this herbal medicine shows good antioxidant potential and may scavenge free radicals and avoid the excessive formation of reactive oxygen species in the body. Whereas the increased oxidative stress plays a significant role in the pathogenesis of DM and multiple complications with the increased formation of free radicals, the antioxidant activity may well add to the mechanisms by which the AoBTHP can be effective in treating diabetes complications [52].

## 4. Materials and Methods

### 4.1. Chemicals and Reagents

Acetic acid, di-sodium hydrogen phosphate dihydrate, ethanol, Folin–Ciocalteu reagent, n-butanol, sodium acetate, sodium carbonate anhydrous, sodium dihydrogen phosphate, and TLC cellulose plates (catalog number 105552) were obtained from Merck^®^ (Darmstadt, Germany). Methanol was obtained from Fisher Chemicals^®^ (Leicestershire, UK). Fast blue B salt was obtained from Fluka^®^ (Buchs, Switzerland). Diphenylboric acid-*β*-ethylamino ester, eosin, gallic acid, glibenclamide, hematoxylin, isoflurane, paraffin, periodic acid, polyethylene glycol-400, protocatechuic acid, Schiff reagent, *α*-glucosidase, *β*-glucosidase, and p-nitrophenyl *α*-D-glucopyranoside were obtained from Sigma-Aldrich^®^ (Steinheim, Germany). 2-naphthyl-*β*-D-glucopyranoside was obtained from Aldrich^®^ (Milwaukee, WI, USA). 2-naphthyl-*α*-D-glucopyranoside was obtained from Glycosynth^®^ (Warrington, UK). Formalin solution, neutral buffered, 10%, was purchased from VWR Chemicals^®^. All used reagents were of analytical grade.

### 4.2. Animals

Male db/db mice (BKS.Cg-+ Leprdb/+ Leprdb/OlaHsd) were purchased from Harlan Laboratories Inc. (Barcelona, Spain). Mice were housed in groups of five or six animals per cage in the animal house of the Faculty of Veterinary Medicine of the University of Lisbon at 20–24 °C and 45–65% relative humidity, under 12–12 h light-dark cycles. Animals had access to standard laboratory chow for diabetic mouse (Teklad Global Diet 2018S; Harlan Laboratories Inc., Barcelona, Spain) and water *ad libitum*. The acclimatization of the animals to laboratory conditions was performed 12 days before the beginning of the study. 

### 4.3. Plant Material

The red type of AoB was manually collected and identified by Luís Catarino, from adult spontaneous plants in Guinea-Bissau. Then, the plant material was dried under shade. Voucher specimens of each sample were deposited at the LISC-Herbarium, Universidade de Lisboa (voucher numbers: AoB collected at Paiai, 11.836° N; 14.421° W: LC 1922 LC).

### 4.4. Extract Preparation

The plant material was homogenized as stated in the European Pharmacopoeia 9.0 [53]. During the study, different portions of the AoBTHP were extemporaneously prepared by macerating the dried AoB in water (1:7 *w*/*v*) for 48 h, between 2 and 8 °C. Then, the extract was filtered using cotton tissue according to the traditional way of preparing the recipe.

### 4.5. HPLC-UV Analysis

The AoBTHP was analyzed with high-performance liquid chromatography (HPLC) using a Waters Alliance 2690/2695 Separations Module (Waters Corporation, Milford, MA, USA) coupled to a Waters 2996 photodiode array detector (UV/DAD) (Waters Corporation, MA). The used column was a Purospher^®^ Star RP-18 end-capped, particle size 5 μm, LiChroCART^®^ 250 × 4 mm, connected to a guard column with the same stationary phase.

Two mobile phases were employed: water + 0.1% formic acid (solvent A) and acetonitrile (solvent B). The flow rate was 0.6 mL/min, and the following gradient solvent was used: from 0 to 13 min → 95:5 (A:B) to 83:17 (A:B), from 13 to 45 min → 83:17 (A:B) to 67:33 (A:B), from 45 to 46 min → 67:33 (A:B) to 60:40 (A:B), from 46 to 50 min ^®^ 60:40 (A:B) to 25:75 (A:B). This was followed by washing and reconditioning of the column. The flow rate of the mobile phase was 0.6 mL/min, and the temperature of the column thermostat was 25 °C. The samples of AoB and the standards were solubilized in methanol (10 mg/mL) and filtered through a polytetrafluoroethylene (PTFE) syringe filter (0.2 μm), before the analysis. 

The chromatogram was monitored with Maxplot (240–650 nm), and data were collected and analyzed using Waters Millennium^®^32 Chromatography Manager (Waters Corporation, MA). The injection volume was 10 μL.

### 4.6. Extract Standardization 

The AoBTHP was standardized based on its total phenolic content, using a modified Folin–Ciocalteu method [54], previously validated by our team [21]. Values were obtained in 3 sets of experiments using a U-2000 Hitachi spectrophotometer (Tokyo, Japan). Results were expressed in milligrams of GAE per gram of dried AoB (mg GAE/g dried AoB) and presented as a mean ± standard error of the mean (SEM). Gallic acid was used as the standard for the calibration curve (y = 0.0086x + 0.1045 and R^2^ = 0.99, where *y* was the absorbance, and *x* were the µg of GAE per µg of AoBTHP).

### 4.7. In Vivo Evaluation of the Hypoglycemic Activity

#### 4.7.1. Experimental Protocol

The experimental protocol of this study was approved by the Ethics and Animal Welfare Committee of the Faculty of Veterinary Medicine of the University of Lisbon. The experiments involving animals were performed according to the requirements of the Directive 2010/63/EU of the European Parliament, and the Council of the European Union [55], and the Portuguese Decree-Law No. 113/2013 [56]. Twenty-six male db/db mice aged 8–11 weeks and weighing 38.32 ± 0.96 g were randomized by age and divided into 5 groups (n = 5 or n = 6). Three doses of the AoBTHP (40.2, 71.5, 127.0 mg/kg/day) were administered, once daily, by oral gavage (10 mL/kg BW). Water was used as the negative control, and glibenclamide (5 mg/kg/day) was used as the positive control. After each administration, signs of morbidity and mortality were monitored. The behavior, body weight, food, and water consumption were monitored daily in each group of animals. On day 92, after the beginning of the treatments, mice were euthanized with a 5% isoflurane inhalation overdose. The terminal blood sampling for biochemical parameters was performed with a cardiac puncture technique, and the body weight was recorded.

#### 4.7.2. Rationale for Dose Selection and Route of Administration

The selected low dose of the AoBTHP (40.2 mg/kg BW/day) corresponded to the traditional human daily dose and was calculated by the Food and Drug Administration conversion factor of animal doses from human equivalent doses [57]. The highest dose of the AoBTHP was 127.0 mg/kg because this was the maximum tolerated dose of the AoBTHP in the repeated dose toxicity test, previously performed by our team [21]. The third dose (71.5 mg/kg) was calculated using the geometric mean of the high and low doses. The choice of that glibenclamide dose of 5 mg/kg/day was made based on published results by other authors [58,59]. All tested samples were orally administered by gavage, the most used route for the administration of drugs used in humans.

#### 4.7.3. Necropsy

All animals were submitted to necropsy after their death. It included the dissection of the dead animals, checkup of the external surface and the thoracic, abdominal, and pelvic compartments, and visualization of the viscera in situ. The liver, kidneys, heart, and pancreas were extracted, and their relative weights were calculated (weight of the organ/body weight × 100).

#### 4.7.4. Biochemical Parameters

During the study, glycemia was measured under non-fasting (in days 0, 24, 43, and 71) or fasting states (in days 8, 31, and 57) using Accu-Chek Aviva^®^ reactive test strips (Roche^®^). Alanine transaminase, aspartate transaminase, cholesterol, serum creatinine, serum urea, and triglycerides were determined in the blood samples that were collected immediately after euthanasia, as described above using a VetTest^®^ chemistry analyzer. 

#### 4.7.5. Histological Analyses

After necropsy, the organs were collected, followed by routine histological processing of the heart, intra-abdominal fat, kidneys, liver, pancreas, and spleen, and subsequent light microscopy observation. After fixation in 10% neutral buffered formalin, the tissues were processed with paraffin, sectioned, and stained with hematoxylin and eosin. All sections were observed under an Olympus BX51 microscope at 40×, 100× and 400× magnification, and microphotographs were obtained with an Olympus digital camera.

### 4.8. In Vitro Evaluation of the Hypoglycemic Activity

#### 4.8.1. α-Glucosidase Inhibitory Assay

Inhibition of *α*-glucosidase was evaluated according to the chromogenic method described by Rouzbehan et al. (2017) [60], with some modifications. The reaction mixture contained 6 μL of *α*-glucosidase (20 unit/mL) and 124 μL of sodium phosphate buffer (pH 6.8, 0.1 M). *p*-nitrophenyl-*α*-D-glucopyranoside (0.01 M) in the same buffer (pH 6.8) was used as the substrate solution. Test samples (20 μL) at various concentrations (100–600 µg/mL) were mixed with enzyme solution in microplate wells and then incubated for 15 min at 37 °C. The reaction was initiated by adding 20 μL of substrate solution and then incubated for an additional 15 min. The reaction was terminated by adding 80 μL of 0.2 M sodium carbonate solution. The absorbance of the wells was measured at 405 nm with the DPC Milenia kinetic analyzer microplate reader. The reaction system without plant extracts was used as the negative control. The acarbose (25–75 mg/mL) was used as the positive control. All determinations were performed in triplicate. The enzyme inhibitory rates of the samples were calculated as follows:

Inhibition % = [(control absorption − sample absorption)/control absorption] × 100(1)

Results were expressed as mean ± standard deviation and presented in in terms of IC_50_ value, which represents the concentration of sample required to inhibit 50% of the enzyme activity.

#### 4.8.2. Detection of α- and β-Glucosidase Inhibitors by Bioautography

TLC bioautographic assays for the detection of α- and *β*-glucosidase inhibitors in the AoBTHP were performed according to the protocol described by Simões-Pires et al. (1990) [61]. Two sets of TLC plates were prepared: one for detection and identification of *α*-glucosidase inhibitors, and other for detection and identification of *β*-glucosidase inhibitors. After migration of a sample of AoBTHP on a TLC cellulose plate using n-butanol-acetic acid-water (4:1:5, v/v/v) as mobile phase, the plate was dried and then sprayed with NEU reagent [62,63]. The TLC was visualized with ultraviolet light (365 nm). Gallic acid was used as the positive control, and its presence in the sample was confirmed using TLC co-chromatography.

### 4.9. Statistical Analysis

Data were analyzed using Microsoft Excel 2013 and GraphPad Prism 5.0. Values are presented as mean ± SEM and were evaluated using the t-test or one-way/two-way analysis of variance (ANOVA), followed by Dunnett’s multiple comparison test or Tukey’s multiple comparison test. Differences were considered statistically significant when the *p*-value was lower than 0.05.

## 5. Conclusions

For the first time, a standardized THP made with stem bark from the red false fruit type of *A. occidentale* was used for an in vivo assessment of hypoglycemic activity, without non-clinical evidence of adverse effects. Blood-glucose lowering activity was observed in this study with AoBTHPs (40.2, 71.5 and 127.0 mg/kg), proving its potential benefits regarding hyperglycemia. The highest dose of the tested AoBTHP showed a more potent glucose-lowering effect than glibenclamide. TLC bioautographic assays showed that hypoglycemic activity of the AoBTHP may be related to its *α*- and *β*-glucosidase inhibition, which is partially due to gallic acid, a major compound in the AoBTHP. 

This study contributed importantly to the validation of the use of the Portuguese AoBTHP as a traditional herbal medicine for type 2 diabetes treatment. 

Further studies are necessary to clarify the mode of action of the tested THP and the contribution of other marker constituents to the verified in vivo hypoglycemic activity. Furthermore, the potential use of such THPs as an add-on to conventional antidiabetic therapy should be explored.

## Figures and Tables

**Table 1:** *A. occidentale* stem bark hypoglycemic activity in vivo studies.

Type of Extraction	Extract Dose and Route of Administration	Duration of Study	Positive Control	Animal Model	Reference
Methanol, Aqueous	100–800 mg/kg *p.o.*	24 h; >24 h	Insulin 5 UI/kg s.c.; glibenclamide 0.2 mg/kg *p.o.*	Streptozotocin 90 mg/kg i.p. Rat	[17]
Hexane	20 and 30 mg/kg i.v.	2 h	-----	Healthy dog	[13]
Methanol	200 mg/kg *p.o.*	21 days	-----	Fructose 25% *w*/*w* in diet; rat	[18]
Hexane	300 mg/kg *p.o.*; 30 mg/kg i.v.	2 h	-----	Healthy dog	[14]
Hydroethanol (80%)	500 mg/kg *p.o.*	28 days	Insulin 5 IU/kg	Streptozotocin 65 mg/kg i.p.; rat	[16]
Ethanol	300 and 500 mg/kg *p.o.* twice daily	28 days	-----	Streptozotocin 65 mg/kg s.c.; rat	[15]

i.p.—intraperitoneal; i.v.—intravenous; *p.o*.—*per os*; s.c.—subcutaneous.

**Table 2:** Weekly mean body weight per group of animals during the study (mean ± SEM).

Week	Negative Control	Positive Control	AoBTHP mg/kg/Day		
			40.2	71.5	127.0
0	40.0 ± 1.8	38.2 ± 2.6	37.9 ± 3.1	38.3 ± 2.0	37.4 ± 1.9
1 to 3	39.4 ± 1.5	36.4 ± 2.7	37.3 ± 2.9	37.2 ± 1.6	36.2 ± 1.8
4 to 6	41.8 ± 2.1	37.2 ± 2.0	38.9 ± 2.6	39.5 ± 1.3	37.5 ± 1.0
7 to 9	43.2 ± 2.6	38.0 ± 1.8	39.9 ± 2.3	39.6 ± 0.8	39.7 ± 1.1
10 to 12	44.8 ± 2.4	39.2 ± 1.9	41.2 ± 2.6	40.1 ± 1.0	40.8 ± 1.2
13	47.2 ± 2.7	41.6 ± 2.2	42.6 ± 2.7	42.1 ± 1.3	41.9 ± 1.1

There were no statistically significant differences between all groups (*p* < 0.05).

**Table 3:** Non-fasting glycemia values of animals (mean ± SEM).

Day	Negative Control	Positive Control	AoBTHP mg/kg/Day		
			40.2	71.5	127.0
0	586 ± 23	582 ± 34	552 ±30	576 ± 27	578 ± 31
24	527 ± 37	486 ± 19 #	524 ± 23	486 ± 15 †	487 ± 9 **
43	591 ± 34	612 ± 21	545 ± 36	565 ± 32	510 ± 17 *
71	633 ± 15	634 ± 14	602 ± 23	618 ± 22	581 ± 21

# *p <* 0.05 vs. Positive control in day 0; † *p <* 0.05 vs. 71.5 mg/kg/day AoBTHP in day 0; * *p <* 0.05 vs. 127.0 mg/kg/day AoBTHP in day 0; ** *p <* 0.01 vs. 127.0 mg/kg/day AoBTHP in day 0.

**Table 4:** Fasting glycemia values of animals (mean ± SEM).

Day	Negative Control	Positive Control	AoBTHP mg/kg/Day		
			40.2	71.5	127.0
8	498 ± 14	491 ± 34	466 ± 18	496 ± 21	545 ± 16
31	540 ± 37	447 ± 9 **	449 ± 39 **	442 ± 30 **	394 ± 18 ****
57	568 ± 28	492 ± 7 *	492 ± 12 *	486 ± 16 *	476 ± 13 **

* *p* < 0.05 vs. negative control; ** *p* < 0.01 vs. negative control; **** *p* < 0.0001 vs. negative control.

**Table 5:** Biochemical parameters of sacrificed animals (mean ± SEM).

Biochemical Parameter	Negative Control	Positive Control	AoBTHP mg/kg/Day		
			40.2	71.5	127.0
ALT (I.U/L)	122 ± 27	119 ± 18	150 ± 54	123 ± 20	139 ± 48
AST (I.U/L)	277 ± 96	320 ± 105	584 ± 158	414 ± 84	338 ± 85
Cholesterol	172 ± 44	134 ± 39	142 ± 29	175 ± 49	149 ± 0.0
Serum creatinine (mg/dL)	0.1 ± 0.0	0.1 ± 0.1	0.4 ± 0.3	0.1 ± 0.1	0.0 ± 0.0
Serum urea (mg/dL)	27 ± 3.0	21 ± 3.0	50 ± 27	27 ± 4.0	21 ± 1.0
Triglycerides (mg/dL)	301 ± 45	282 ± 57	323 ± 52	225 ± 65	257 ± 41

ALT: alanine transaminase; AST: aspartate transaminase. There were no statistically significant differences among all groups (*p* < 0.05).

**Table 6:** Relative organ weights collected for each group of animals sacrificed—percentage g/g of body weight (mean ± SEM).

Organ	Negative Control	Positive Control	AoBTHP mg/kg/Day		
			40.2	71.5	127.0
Heart	0.24 ± 0.01	0.28 ± 0.01	0.20 ± 0.06	0.28 ± 0.01	0.25 ± 0.01
Kidneys	0.90 ± 0.03	1.05 ± 0.06	0.96 ± 0.09	0.88 ± 0.04	0.87 ± 0.02
Liver	6.24 ± 0.17	6.10 ± 0.14	5.63 ± 0.51	5.74 ± 0.38	5.57 ± 0.29
Pancreas	0.42 ± 0.06	0.39 ± 0.12	0.32 ± 0.05	0.38 ± 0.03	0.35 ± 0.03
Spleen	0.18 ± 0.03	0.10 ± 0.01	0.10 ± 0.01	0.15 ± 0.03	0.10 ± 0.01

There were no statistically significant differences among all groups (*p* < 0.05).

## Data Availability

Not applicable.
